# Mice lacking the synaptic adhesion molecule Neph2/Kirrel3 display moderate hyperactivity and defective novel object preference

**DOI:** 10.3389/fncel.2015.00283

**Published:** 2015-07-28

**Authors:** Su-Yeon Choi, Kihoon Han, Tyler Cutforth, Woosuk Chung, Haram Park, Dongsoo Lee, Ryunhee Kim, Myeong-Heui Kim, Yeeun Choi, Kang Shen, Eunjoon Kim

**Affiliations:** ^1^Department of Biological Sciences, Korea Advanced Institute of Science and TechnologyDaejeon, South Korea; ^2^Department of Neuroscience and Division of Brain Korea 21, Biomedical Science, College of Medicine, Korea UniversitySeoul, South Korea; ^3^Department of Neurology, Columbia University Medical CenterNew York, NY, USA; ^4^Department of Biomedical Sciences, Graduate School of Medical Science and Engineering, Korea Advanced Institute of Science and TechnologyDaejeon, South Korea; ^5^Center for Synaptic Brain Dysfunctions, Institute for Basic ScienceDaejeon, South Korea; ^6^Department of Biology, Stanford UniversityStanford, CA, USA; ^7^Howard Hughes Medical InstituteChevy Chase, MD, USA

**Keywords:** synaptic adhesion, intellectual disability, cognition, autism spectrum disorder, hyperactivity, memory, synaptic transmission

## Abstract

Synaptic adhesion molecules regulate diverse aspects of neuronal synapse development, including synapse specificity, formation, and maturation. Neph2, also known as Kirrel3, is an immunoglobulin superfamily adhesion molecule implicated in intellectual disability, neurocognitive delay associated with Jacobsen syndrome, and autism spectrum disorders. We here report mice lacking Neph2 (*Neph2^-/-^* mice) display moderate hyperactivity in a familiar, but not novel, environment and defective novel object recognition with normal performances in Morris water maze spatial learning and memory, contextual fear conditioning and extinction, and pattern separation tests. These mice also show normal levels of anxiety-like behaviors, social interaction, and repetitive behaviors. At the synapse level, *Neph2^-/-^* dentate gyrus granule cells exhibit unaltered dendritic spine density and spontaneous excitatory synaptic transmission. These results suggest that Neph2 is important for normal locomotor activity and object recognition memory.

## Introduction

Synaptic adhesion molecules play important roles in the regulation of synapse specificity, formation, maturation, and plasticity (Yamagata et al., 2003; Dalva et al., 2007; Biederer and Stagi, 2008; Sudhof, 2008; Brose, 2009; Woo et al., 2009; Johnson-Venkatesh and Umemori, 2010; Shen and Scheiffele, 2010; Tallafuss et al., 2010; Williams et al., 2010; de Wit et al., 2011; Nam et al., 2011; Siddiqui and Craig, 2011; Yuzaki, 2011; Wright and Washbourne, 2011; Krueger et al., 2012; Missler et al., 2012; Valnegri et al., 2012; Song and Kim, 2013; Takahashi and Craig, 2013; Um and Ko, 2013; de Wit and Ghosh, 2014; Yogev and Shen, 2014). A large number of synaptic adhesion molecules with these functions have been reported, including the prototypical pair of neuroligins and neurexins (Sudhof, 2008).

Neph2 belongs to the Ig domain-containing family of adhesion molecules (Sellin et al., 2003; Yogev and Shen, 2014), and contains three known members, Neph1, Neph2, and Neph3 (also known as Kirrel, Kirrel3, and Kirrel2). Neph2 was originally identified as a junctional component in the slit diaphragm of the glomerulus (Sellin et al., 2003), but has also been found to be strongly expressed in the brain (Gerke et al., 2005). Structurally, Neph2 contains five Ig-like domains in the extracellular region, followed by a single transmembrane domain, and a PDZ-binding motif at the C-terminus. Neph2 proteins interact homophilically with each other, and heterophilically with nephrin (Gerke et al., 2005), another adhesion molecule in the Ig superfamily.

Functionally, SYG-1, a Caenorhabditis elegans homolog of Neph2, determines where the nerve terminals of the HSN motor axons should be localized, by interacting with SYG-2 displayed on target epithelial cells (Shen and Bargmann, 2003; Shen et al., 2004; Yogev and Shen, 2014). Two related proteins in Drosophila, IrreC/Rst and Kirre regulate the development of eyes and muscles and the projection of visual axons (Ramos et al., 1993; Strunkelnberg et al., 2001; Bao and Cagan, 2005). In mammals, Neph2 regulates axon sorting and targeting in the olfactory bulb (Serizawa et al., 2006), and the formation of the pontine nucleus in the developing hindbrain, likely through the organization of migratory behaviors of pontine nucleus neurons in the presumptive nuclear region (Nishida et al., 2011). In addition, Neph2 regulates the coalescence of sensory neuronal axons in the vomeronasal system (Prince et al., 2013). These results suggest that Neph2 regulates axonal sorting, migration, and targeting during mammalian brain development. However, the potential role of Neph2 in synapse development has not been explored, except for the finding that Neph2 directly interacts with CASK (Gerke et al., 2006), a synaptic scaffolding protein implicated in synapse development and brain disorders such as X-linked microcephaly and intellectual disability (ID; Hsueh, 2006, 2009; Moog et al., 2011).

Clinically, previous studies have implicated Neph2 in mild to severe ID (Bhalla et al., 2008), neurocognitive delay associated with Jacobsen syndrome (Guerin et al., 2012), and autism spectrum disorders (ASDs; Talkowski et al., 2012; Cheng et al., 2013). These results implicate Neph2 in brain development and related disorders, although detailed mechanisms remain largely unexplored.

In order to explore the functions of Neph2 in synapse development and related behaviors, we used *Neph2^-/-^* mice to perform various behavioral assays combined with biochemical, cell biological, and electrophysiological characterizations. Our main focus for the present study was the hippocampus because the Neph2-related disorders, including ID, Jacobsen syndrome, and ASDs, commonly involve learning and memory deficits, which are in turn routinely associated with the hippocampus. Our results indicate that *Neph2^-/-^* mice are slightly hyperactive in a familiar but not in a novel environment. In addition, these mice display defective novel object preference, although other types of learning and memory behaviors tested are largely normal.

## Materials and Methods

### Antibodies

GST-fusion protein containing human Neph2 (aa 563–778) and synthetic peptide mimicking the last 10 aa of human Neph2 were used to immunize rabbits (1344 and 1468, respectively). For CaMKIIα/β polyclonal antibodies, GST-fusion proteins containing full-length CaMKIIα were used to immunize guinea pigs (Gp). The following antibodies have been described: EGFP (1173, Rb; Ko et al., 2003), PSD-95 (1402, Gp), SAP102 (1447, Gp) (Choi et al., 2005), PSD-93 (1634, Rb), SAP97 (1443, Gp) (Oh et al., 2010), CASK (1640, Rb), GluA1 (1193, Rb), GluA2 (1195, Rb) (Kim et al., 2009). The following antibodies were purchased: synapsin I (Chemicon), synaptophysin (Santa Cruz), GluN2A (Invitrogen), GluN2B (BD Transduction Lab), mGluR5 (Millipore), PAK1/3, p-PAK1/3, LIMK1, p-LIMK1, Cofilin, p-Cofilin, and ROCK1 (Cell Signaling), α-Tubulin (Sigma), and NeuN (Millipore).

### Animals

*Neph2^-/-^* mice have been reported recently (Prince et al., 2013). *Neph2^-/-^* mice were maintained in the C57BL6/J background, and all mice used in experiments were obtained by mating heterozygous mice. Mice were bred and maintained according to the Requirements of Animal Research at KAIST, and all procedures were approved by the Committee of Animal Research at KAIST (KA2012-19). Mice were fed *ad libitum*, and 2–6 animals were housed in a cage under 12-h light-dark cycles.

### Slice Immunohistochemistry

Mouse brain sections (2–3 weeks) were prepared by cardiac perfusion (4% paraformaldehyde). After 12-h post-fixation, 50 μm brain sections were prepared using a vibratome (VT1000S, Leica). Brain sections were washed three times with PBS for 10 min, permeabilized with 0.5% TritonX-100 for 30 min, blocked with 5% bovine serum albumin (BSA) for 1 h, stained with primary antibodies at 4°C for 12 h, stained with secondary antibodies for 1 h, and mounted using Vectashield. For visualization of EGFP expression, a confocal microscope (x 10 objective; LSM780, Carl Zeiss) was used to capture the images and stitch them to make large composite images.

### Electrophysiology

For slice preparation, following ethyl ether anesthetization of wild-type (WT) or *Neph2^-/-^* mice (P49–56), brains were removed and sliced in sagittal sections (300 μm) across the dorsal hippocampus in a (5% CO_2_) carbogen-bubbled, ice-cold sucrose cerebral spinal fluid (sCSF) consisting of–in mM–212 sucrose, 25 NaHCO_3_, 5 KCl, 1.25 NaH_2_PO_4_, 10 D-glucose, 2 Na-pyruvate, 1.2 Na-ascorbate, 3.5 MgCl_2_, 0.5 CaCl_2_ using a vibratome (VT1200S, Leica). Slices were recovered at 32°C for 15 min in artificial cerebral spinal fluid (aCSF) consisting of–in mM–125 NaCl, 25 NaHCO_3_, 2.5 KCl, 1.25 NaH_2_PO_4_, 10 D-glucose, 1.3 MgCl_2_, 2.5 CaCl_2_ and maintained thereafter at room temperature.

Recordings were made by using Multiclamp 700B amplifier (Molecular Devices) under visual control with differential inference contrast illumination in an upright microscope (BX50WI, Olympus) and were filtered at 2 kHz and digitized at 10 kHz. Series resistance was monitored and any data with resistance greater than 20 MΩ were discarded. Data were acquired via Clampex 9.2 (Molecular Devices) and analyzed by Clampfit 9 (Molecular Devices) or using custom macros written in Igor (Wavemetrics).

To record mEPSCs, hippocampal sections were perfused with aCSF containing 0.5 μM tetrodotoxin and 60 μM picrotoxin, and cells were voltage clamped at -70 mV. Recording pipettes of 3–3.8 MΩ resistance were, for all whole cell recordings unless otherwise stated, filled with a Cs gluconate-based internal solution of 280–290 mOsm (pH 7.3) containing–in mM–110 Cs gluconate, 8 NaCl, 10 TEA-Cl, 20 HEPES, 5 Qx-314Cl, 4 Mg-ATP, 0.3 Na-GTP, 0.5 EGTA. For biocytin injection and spine analysis, 0.2% biocytin was added to the aforementioned internal solution.

### Spine Analysis

Spine density was measured by injecting biocytin into dentate gyrus (DG) granule cells from WT and *Neph2^-/-^* mice. Spine images were captured by confocal microscope (LSM780, Carl Zeiss) and analyzed using MetaMorph image analysis software (Universal Imaging). For spine quantification, minimum two dendrites of a single granule cell were randomly selected. The numbers of spines on each dendrite from a single cell were averaged. Dendritic protrusions shorter than 0.5 μm or longer than 3.0 μm were not counted as spines.

### Behavioral Tests

The behavioral tests described below were performed in the order of handling, automated 24-h movement, open field, novel object recognition, elevated plus maze, self-grooming, three-chamber social interaction, and Morris water maze or contextual fear conditioning/extinction. In another set, behavioral tests were performed in the order of handling, buried food test, radial arm maze, and contextual discrimination. Intervals between each behavioral test were 1–3 days, except for the 2-weeks interval between radial arm maze and contextual discrimination tests, which was to ensure body weight recovery and sufficient rest.

### Open Field Test

The size of the open field box was 40 cm × 40 cm × 40 cm, and the center zone line was 10 cm apart from the edge. The testing room was illuminated at ∼20 lux or in complete darkness. Mice were placed in the center of the box in the beginning of test, and mouse movements were recorded with a video camera for 60 min, and analyzed using EthoVision XT 10 program (Noldus).

### Automated 24-h Movement Analysis

For a long-term and real-time movement analysis, we used the Laboras system (Metris), designed to detect and analyze vibrations delivered from a cage to a carbon-fiber vibration-sensitive plate below the cage for mouse movements including locomotion, grooming, rearing, and climbing. Before the data collection, mice were isolated and habituated for a week in the same room with the Laboras set up. After the habituation, each mouse was placed in the cage, and data were collected uninterrupted for 24 h. Collected data were analyzed by the Laboras software.

### Elevated Plus Maze Test

The elevated plus maze consisted of two open arms, two closed arms, and a center zone, and elevated to a height of 50 cm above the floor. Mice were placed in the center zone and allowed to explore the space for 10 min. The data was analyzed by EthoVision XT 10 program, automatically.

### Three-Chamber Social Interaction Test

The size of three-chambered apparatus was 40 cm W × 20 cm H × 26 cm D with a center chamber of 12 cm W and side chambers of 14 cm W. Both side chambers contained a plastic cage in the corner, with a plastic cup with a weight on it, to prevent the subject mouse from climbing. The assay consisted of four sessions. The first session began with 10 min habituation in the center chamber followed by the second 10 min session where the subject mouse could freely explore all three chambers. The mouse was then gently confined in the center chamber while a novel ‘Object’ and a WT stranger mouse ‘Stranger 1’ was placed in the two plastic cages. The subject mouse was then allowed to freely explore all three chambers for 10 min. Before the last session, the subject mouse was again gently guided to the center chamber while the ‘Object’ was replaced with a WT ‘Stranger 2’ mouse. The subject mouse again freely explored all three chambers for 10 min. All stranger mice were males at the same age and habituated to the plastic cage a day before the test for 30 min. The positions of ‘Object’ and ‘Stranger 1’ were alternated between tests to prevent side preference.

### Buried Food Test

Before the experiments, mice were restricted in their diet to maintain 80–85% of their natural weight by being supplied with just 1–2 g of foods. Two types of food were used for the test mouse olfaction, usual food pellet and vanilla-flavored cookies. The cages with bedding (3 cm deep) were buried of food at sites 1.5 cm apart from the cage wall. Mice on diet restriction for 5 days were placed in the cage and allowed to explore the hidden food to measure the time taken to find food.

### Self-Grooming

This test was performed in a standard home cage without bedding to limit natural digging behaviors of mice. Mice were individually placed in a home cage during 10 min, and their behavior was recorded and analyzed by an observer blind to the identity of subject mice. Grooming behavior was defined as stroking or scratching of face, head, or body with the two forelimbs, or licking body parts.

### Novel Object Recognition Test

Object recognition test was performed in the open field box. Mice were habituated to the open field box for 20 min 24 h before the sample phase. During the sample phase, mice were allowed to explore two identical objects for 10 min. Exploration time for each object was measured. Test phase, where one of the two objects was replaced with a new one, was performed 24 h later, and exploration time for each object was measured. Object exploration was defined as each instance in which a mouse’s nose touched the object or was oriented toward the object and came within 2 cm of it.

### Morris Water Maze Test

Mice were trained to find the hidden platform (10 cm diameter) in a white plastic tank (120 cm diameter). Mice were given three trials per day with an inter-trial interval of 30 min. The learning phase of the water maze was performed for seven consecutive days, followed by the probe test on day 8 where mice were given 1 min to find the removed platform. For reversal training (days 9–11), the location of the platform was switched to the opposite position from the previously trained one, and mice were trained to learn the new position of platform. Target quadrant occupancy and the exact crossing over the former platform location during the probe test were measured by EthoVision 3.1 program.

### Contextual Fear Conditioning and Extinction

All experiments were carried out in a fear conditioning system (Coulbourn Instruments). Training and testing were performed in a Plexiglas chamber with a stainless steel grid floor with constant illumination (50 lux). The chamber was cleaned with 70% ethanol before each session. On the first day of test, mice were pre-exposed in the fear conditioning chamber for 5 min. On the next day, they were given 0.5 mA of foot-shock for 2 s for three times: at 2-, 3-, and 4-min time points and taken out from the chamber at 5 min. Twenty four hours after the training, mice were re-exposed to the chamber for 5 min without foot shock, and freezing levels were measured for the initial 2 min. For fear extinction, mice received foot-shock on day 1 and were repeatedly exposed to the chamber without shock during days 2–6.

### Contextual Discrimination

We subjected mice to a contextual fear conditioning test using a less distinct pair of contexts (A and B) that shared an identical metal grid floor, but had unique wall-paper, bottom color, and lighting. In this protocol, fear conditioning took place incrementally over several days in order to investigate the effects of repeated experiences. The experiments were performed in the same condition of time, temperature, and humidity: 9:00 am–4:00 pm, 20–22°C, and 45%. Each trial was performed after habituation in their cage for 30–60 min and twice a day at 9:00 am and 4:00 pm. During days 1–3 of the experiment, mice were placed in a shock chamber for 180 s, given a single foot-shock of 0.65 mA for 2 s, and taken out after 60 s. On days 4 and 5, for generalization, mice of each genotype were divided into two groups, one visiting chamber A and the other visiting chamber B (one trial/day) on day 4, and visiting the unvisited chamber on day 5. Mice did not receive foot-shock during the generalization, and freezing was assessed. During days 6–11, for discrimination training, mice visited the two chambers every day for 6 days, always receiving a foot-shock 180 s after being placed in chamber A but not chamber B. Freezing during the first 3-min exposure in each chamber was used to calculate a daily discrimination ratio.

### Radial Arm Maze Test

Mice were diet restricted to reach 80–85% of weight for 5 days, during which mouse handling was performed. Each arm maze test was preceded by habituation of the mice in home cages to the room with the radial arm maze apparatus with constant temperature (19–22°C), humidity (30–40%), and brightness (80–170 lux) for an hour. Mice were then habituated to the radial arm maze apparatus, by putting them with cage mates in the apparatus with reward milk (50 μl) on every well for 1 h, followed by individual mouse habituation for 1 h. In the sample phase, a mouse was placed in the start arm and allowed to explore the sample arm with reward milk (30 μl) for 60 s, during which the choice arm was closed. We removed the mice from the apparatus when they finished eating the reward milk, spent more than 10 s in the sample arm, or moved out the sample arm. Fifteen seconds after the sample phase, mice were subjected to the choice phase, by putting a mouse at the start arm and giving two choices, previously visited incorrect arm and unvisited correct arm (with reward milk). The angle between sample and choice arms were randomly selected.

### Statistics

Statistical details are described in Supplementary Table S1.

## Results

### General Characterization of the *Neph2^-/-^* Brain

**FIGURE 1:**
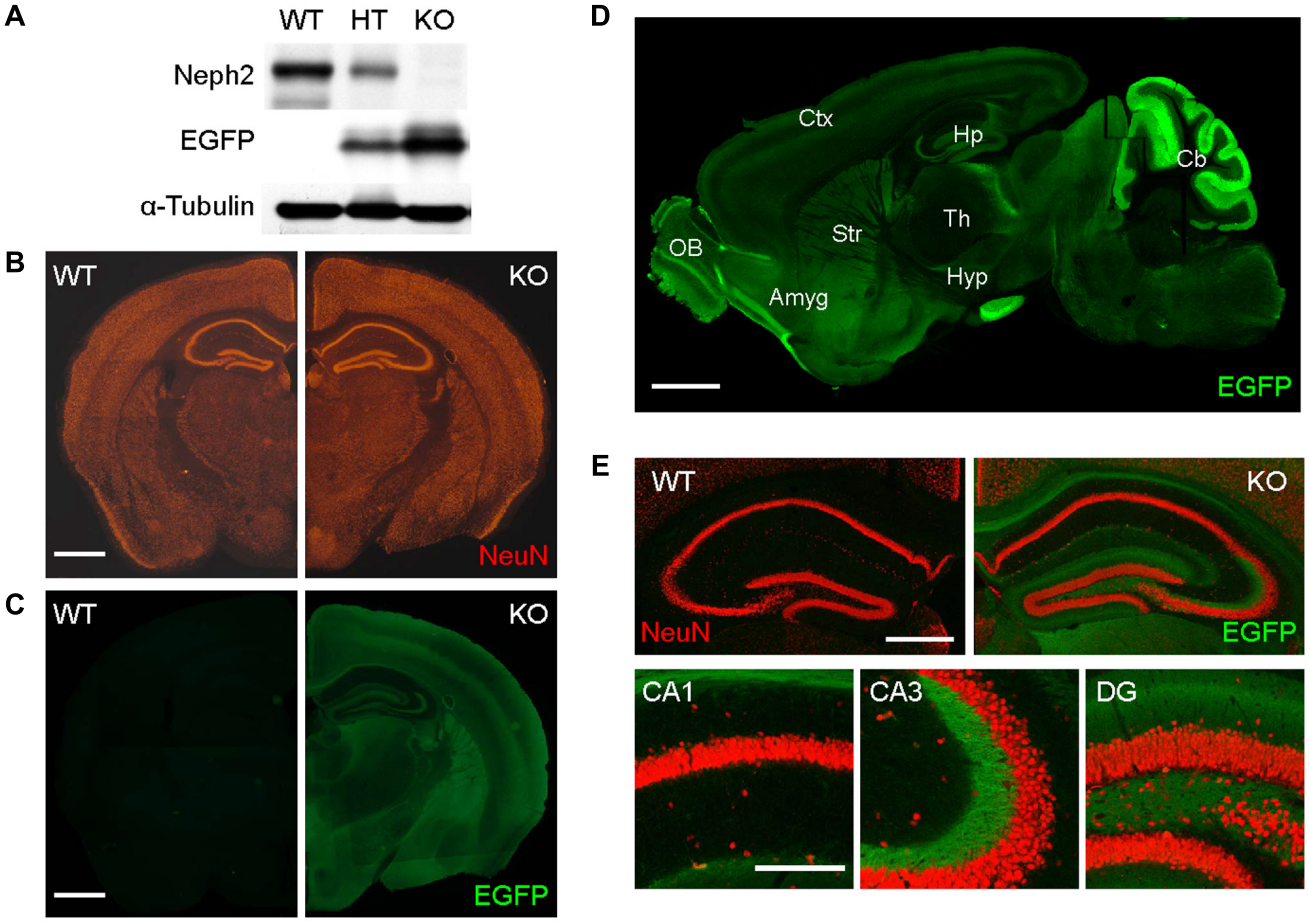
**Normal gross morphology of the *Neph2^-/-^* brain. (A)** Absence of Neph2 protein detection in the *Neph2^-/-^* brain. WT, *Neph2^+/-^* (*HT*), and *Neph2^-/-^* (*KO*) whole brain lysates (3 weeks) were immunoblotted with Neph2 (1468 rabbit) antibodies. Note that EGFP expression driven by the endogenous Neph2 promoter is higher in the *Neph2^-/-^* brain, relative to *Neph2*^+^*^/-^* brain. **(B)** Normal gross morphology of the *Neph2^-/-^* brain (3 weeks), revealed by NeuN (neuron-specific marker) staining. Scale bar, 0.5 mm. **(C–E)** Expression patterns of Neph2 in the brain, revealed by EGFP expression driven by the endogenous Neph2 promoter. *Neph2^-/-^* coronal **(C,E)** and sagittal **(D)** brain sections (3 weeks) were doubly stained for EGFP and NeuN. **(E)** Shows further details of Neph2 expression in the hippocampal coronal section. Ctx, cortex; Hp, hippocampus; Str, striatum; Th, thalamus; OB, olfactory bulb; Amyg, amygdala; Hyp, hypothalamus; Cb, cerebellum. Scale bar, 0.5 mm for **(B–D)** and 0.2 mm for **(E)**.

In order to explore Neph2 functions *in vivo*, we analyzed *Neph2^-/-^* (or *Kirrel3^-/-^*) mice, which has recently been reported to display impaired coalescence of vomeronasal axons and a loss of male–male aggression (Prince et al., 2013). The *Neph2^-/-^* brain showed no detectable Neph2 proteins (**Figure 1A**) and largely normal gross morphology of the brain, as revealed by NeuN staining (neuron-specific marker; **Figure 1B**). EGFP expression driven by the endogenous Neph2 promoter revealed that Neph2 is expressed in diverse brain regions, including the cortex, hippocampus, striatum, olfactory bulb, and cerebellum (**Figures 1C,D**; Supplementary Figures S1 and S2).

In the hippocampus, EGFP signals were mainly detected in the dendritic field of the DG and mossy fiber axons projecting to the CA3 region, but little in CA3 or CA1 dendrites (**Figure 1E**), suggesting that DG granule cells are the prominent site of Neph2 expression in the hippocampus.

**FIGURE 2:**
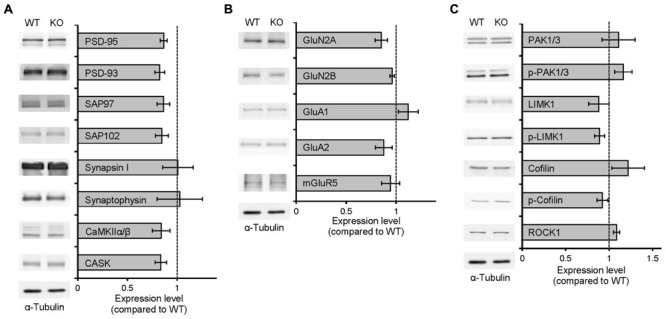
**Normal levels of synaptic proteins and actin-regulatory proteins in *Neph2^-/-^* mice. (A–C)** *Neph2^-/-^* brains show normal levels of synaptic scaffolding proteins **(A)**, signaling proteins **(B)**, and levels and activity (shown by protein phosphorylation) of actin regulatory proteins **(C)**. *Neph2^-/-^* total hippocampal cell lysates (3 weeks) were immunoblotted with the indicated antibodies. (*n* = 4 for WT and KO).

Neph2 deletion did not cause any changes in the expression levels of synaptic scaffolding, signaling, and receptor proteins tested in the hippocampus, nor on the levels and activity (protein phosphorylation) of actin regulatory proteins (**Figures 2A–C**).

### *Neph2^-/-^* Mice Display Hyperactivity in a Familiar Environment but Normal Anxiety-Like Behavior and Social Interaction

**FIGURE 3:**
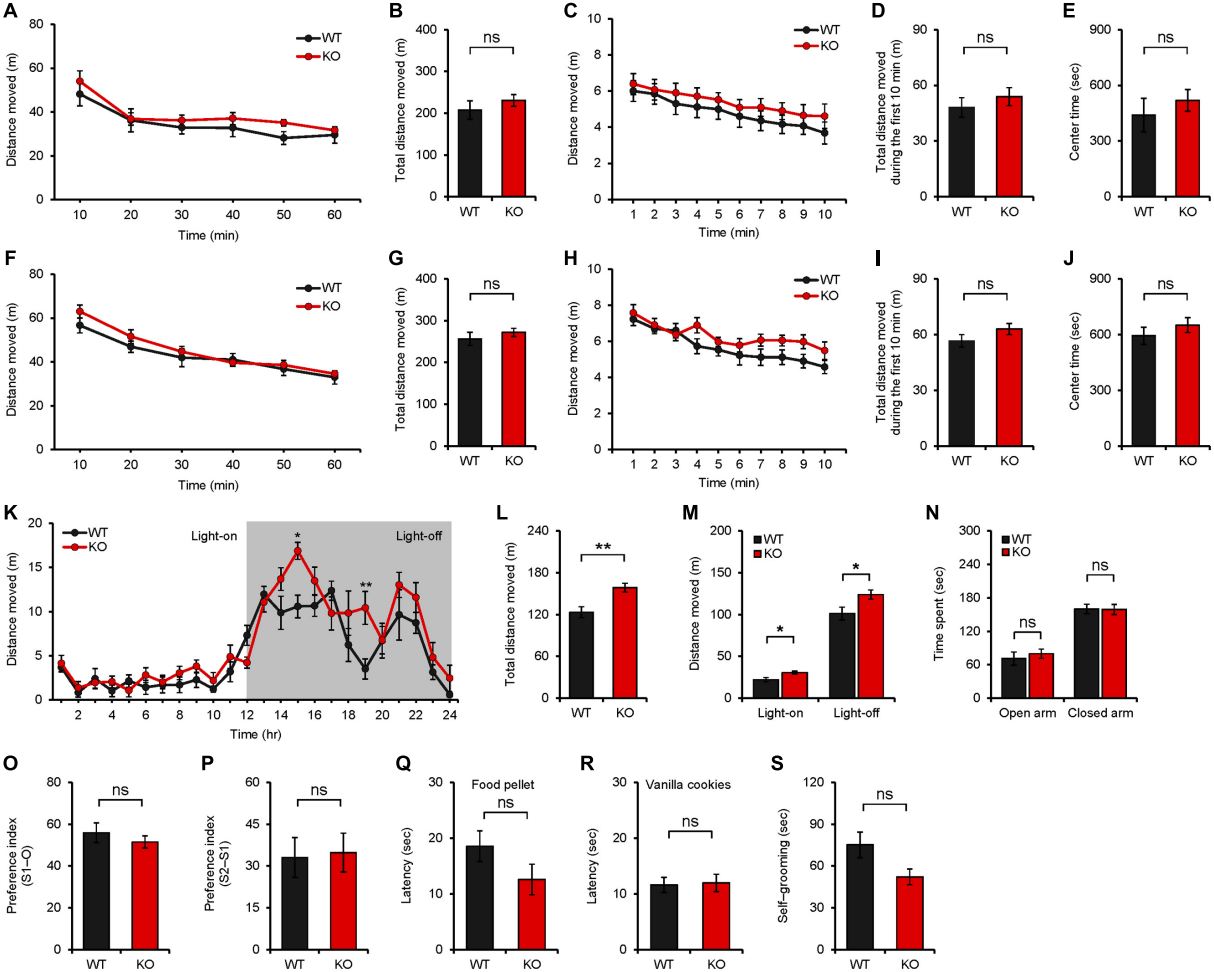
***Neph2^-/-^* mice display enhanced locomotion in a familiar environment but normal anxiety-like behavior and social interaction. (A–J)** *Neph2^-/-^* mice (8–16 weeks) show normal locomotor activities in a novel environment, as measured by the open field test performed at ∼20 lux **(A–E)** and in complete darkness **(F–J)**. Locomotor activities during the first 10 min are also indicated **(C,D,H,I)**. Time in the center region of the open field arena **(E,J)** was analyzed to measure anxiety-like behavior. (*n* = 8 for WT, and 9 for KO for 20 lux, and 8 for WT and KO for complete darkness, ns, not significant, repeated measures ANOVA and Student’s *t*-test). **(K–M)** *Neph2^-/-^* mice (8–16 weeks) show enhanced locomotion in a familiar environment, as measured by 24-h continuous monitoring of mouse movements in a home cage-like environment (Laboras cage; see Materials and Methods for details). Both total distance moved (light-on + light-off phase) **(L)** and the distance moved during light-on or light-off phase **(M)** are indicated. (*n* = 7 for WT, and 9 for KO, ^∗^*p* < 0.05, ^∗∗^*p* < 0.01, repeated measures ANOVA and Student’s *t*-test). **(N)** Normal anxiety-like behavior of *Neph2^-/-^* mice (8–16 weeks) in the elevated plus maze test. (*n* = 6 for WT, and 8 for KO, ns, not significant, Student’s *t*-test). **(O,P)** Normal social interaction and social novelty recognition of *Neph2^-/-^* mice in the three-chamber test. The social preference index represents the numerical difference between the times spent sniffing the two targets (S1/stranger versus O/object or S2/new stranger versus S1/previous stranger) divided by total time spent × 100. (*n* = 12 for WT, and 15 for KO, ns, not significant, Student’s *t*-test). **(Q,R)** Normal olfactory function of *Neph2^-/-^* mice in food and cookie retrieval assays. (*n* = 12 for WT, and 8 for KO for food pellet and vanilla cookies, ns, not significant, Student’s *t*-test). **(S)** Normal self-grooming of *Neph2^-/-^* mice in home cages without bedding. (*n* = 7 for WT and KO, ns, not significant, Student’s *t*-test).

We next tested whether *Neph2^-/-^* mice (8–16 weeks) display any alterations in behaviors. *Neph2^-/-^* mice showed normal locomotor activity in a novel environment, as measured by the open field test performed at ∼20 lux (**Figures 3A–D**). Another open field test performed in complete darkness yielded similar results (**Figures 3F–I**). Intriguingly, *Neph2^-/-^* mice displayed enhanced locomotor activity in their home cages during the light-off phase (see Materials and Methods for details), as determined by 24-h continuous monitoring of locomotor activities (**Figures 3K–M**). This suggests that *Neph2^-/-^* mice show normal locomotion in the novel environment but hyperactivity in the familiar environment.

*Neph2^-/-^* mice spent a normal amount of time in the center region of the open field area (**Figures 3E,J**), suggesting that they do not display anxiety-like behavior. Consistently, *Neph2^-/-^* mice spent a comparable amount of time in the open or closed arms of the elevated plus maze, as compared with WT mice (**Figure 3N**).

In experiments exploring autistic-like behaviors, *Neph2^-/-^* mice displayed normal social interaction and social novelty recognition in the three-chamber test (**Figures 3O,P**). The olfactory functions of *Neph2^-/-^* mice were unaffected (**Figures 3Q,R**). In assays measuring repetitive behavior, *Neph2^-/-^* mice displayed levels of self-grooming comparable to those in WT mice (**Figure 3S**).

### *Neph2^-/-^* Mice Display Impaired Novel Object Recognition but Normal Spatial and Fear Memory

**FIGURE 4:**
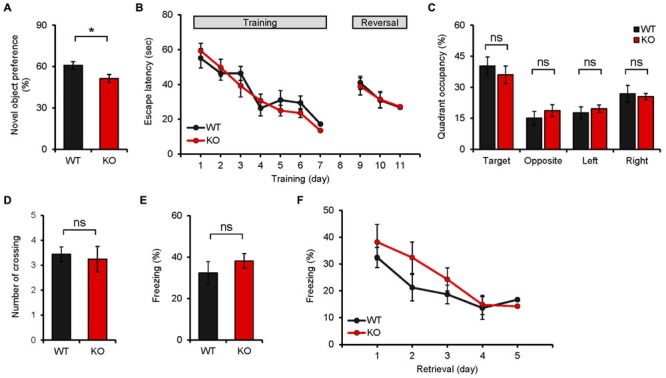
***Neph2^-/-^* mice display reduced novel object recognition but normal spatial and fear learning and memory. (A)** *Neph2^-/-^* mice (8–16 weeks) show reduced novel object recognition. (*n* = 12 for WT, and 15 for KO, ^∗^*p* < 0.05, Student’s *t*-test). **(B–D)** *Neph2^-/-^* mice (8–16 weeks) perform normally in the learning, probe, and reversal learning phases of the Morris water maze test. The number of exact crossing over the platform **(D)** represents a more stringent measure of spatial memory. The swimming speed was also normal (data not shown). (*n* = 9 for WT and KO, ns, not significant, repeated measures ANOVA and Student’s *t*-test). **(E)** Normal contextual fear conditioning in *Neph2^-/-^* mice (8–16 weeks). (*n* = 9 for WT and KO, ns, not significant, Student’s *t*-test). **(F)** Normal contextual fear extinction in *Neph2^-/-^* mice. (*n* = 9 for WT and KO, ns, not significant, Student’s *t*-test).

We next explored learning and memory phenotypes of *Neph2^-/-^* mice (8–16 weeks), given that the DG region, where Neph2 is highly expressed, is a part of the hippocampus that contributes to many types of learning and memory behaviors. In the novel object recognition test, where mice were exposed to two identical objects on the first day and given a new object that replaces one of the two familiar objects on the second day, *Neph2^-/-^* mice largely failed to recognize the new object, contrary to WT mice (**Figure 4A**). In the Morris water maze test, *Neph2^-/-^* mice performed normally during the learning, probe, and reversal learning phases (**Figures 4B–D**). These results suggest that *Neph2^-/-^* mice have impaired object recognition memory but normal spatial learning and memory.

In the contextual fear conditioning test, *Neph2^-/-^* mice showed fear levels comparable to those of WT mice when re-exposed to the same environment 24 h after the training (**Figure 4E**). In addition, *Neph2^-/-^* mice displayed fear extinction levels similar to those of WT mice, when fear-conditioned mice were exposed to the same context for five consecutive days without foot shock (**Figure 4F**).

### *Neph2^-/-^* Mice Display Normal Pattern Separation

**FIGURE 5:**
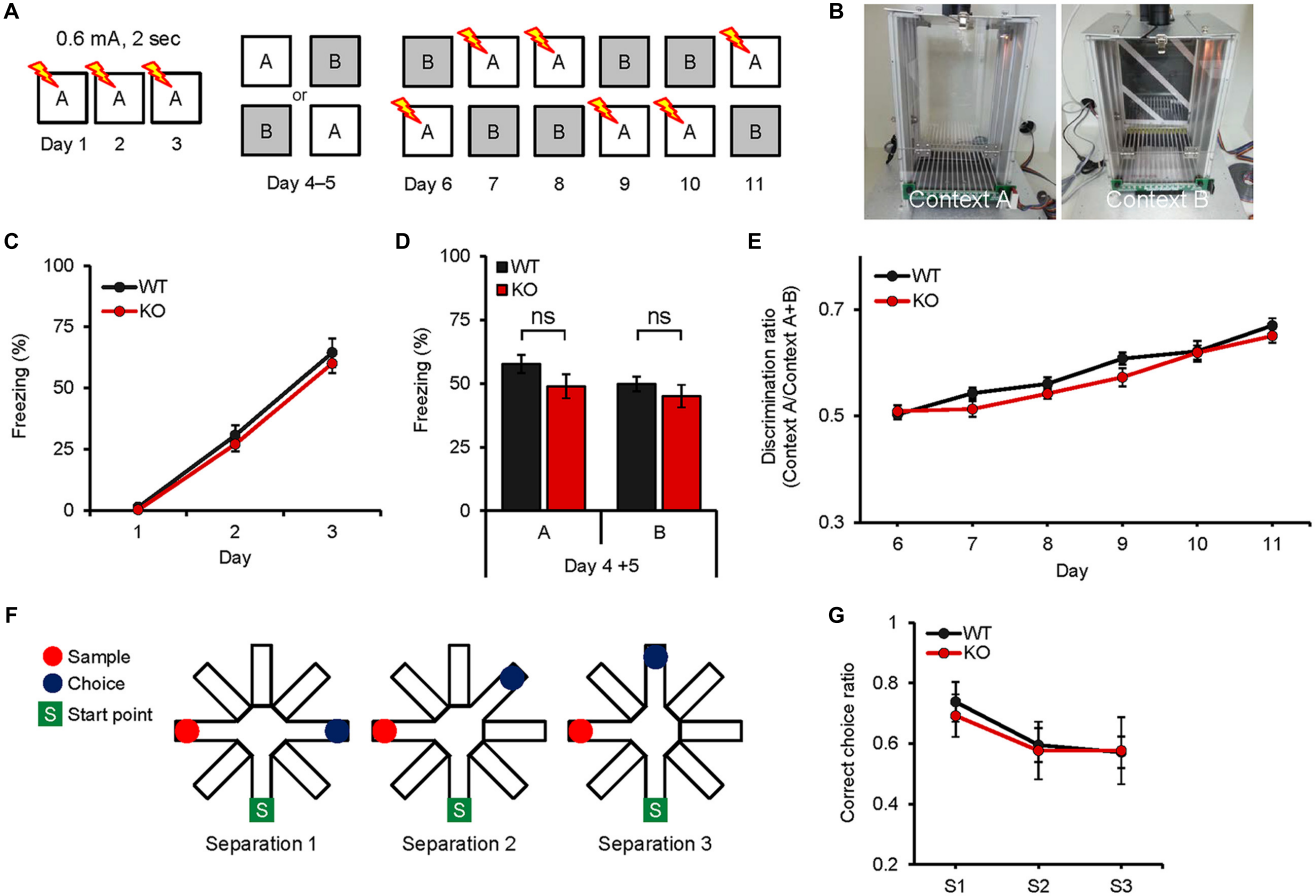
***Neph2^-/-^* mice display normal pattern separation. (A–E)** Normal pattern separation in *Neph2^-/-^* mice (8–16 weeks), as determined by the fear conditioning test using two similar contexts. Freezing levels during stages of fear conditioning (days 1–3), introduction of a similar context (context B; ays 4–5), and discrimination learning of contexts A and B (days 6–11) are indicated. (*n* = 10 for WT and KO, ns, not significant, Student’s *t*-test and repeated measures ANOVA). **(F,G)** Normal pattern separation of *Neph2^-/-^* mice (8–16 weeks) in the radial arm maze working memory test, where three different arm angles (sample vs. choice) were used. (*n* = 21 for WT, and 13 for KO, repeated measures ANOVA).

Because the DG, where Neph2 is highly expressed, has been implicated in differentiating similar contexts (Yassa and Stark, 2011), we subjected *Neph2^-/-^* mice to the following two different pattern separation assays. In the first assay, *Neph2^-/-^* mice fear-conditioned in the context A (days 1–3) were exposed to the same context A or a similar context B (day 4–5), followed by exposures to the context A combined with shock, or the context B without shock, for 6 days (days 6–11) to associate the context A with foot-shock (**Figures 5A,B**). *Neph2^-/-^* mice acquired this associative fear memory normally during days 6–11, showing increasing levels of freezing in the context A relative to B that were comparable to those in WT mice (**Figures 5C–E**).

In the second assay, we measured pattern separation under the context of working memory using the radial arm maze. When *Neph2^-/-^* mice were subjected to a working memory test in which mice have to remember the unvisited arm in order to find a food reward, *Neph2^-/-^* mice showed working memory levels comparable to those in WT mice (**Figures 5F,G**). These results collectively suggest that *Neph2^-/-^* mice have normal capability to separate similar patterns on par with WT mice.

### *Neph2^-/-^* Mice Show Normal Levels of Spine Density and Spontaneous Excitatory Synaptic Transmission

**FIGURE 6:**
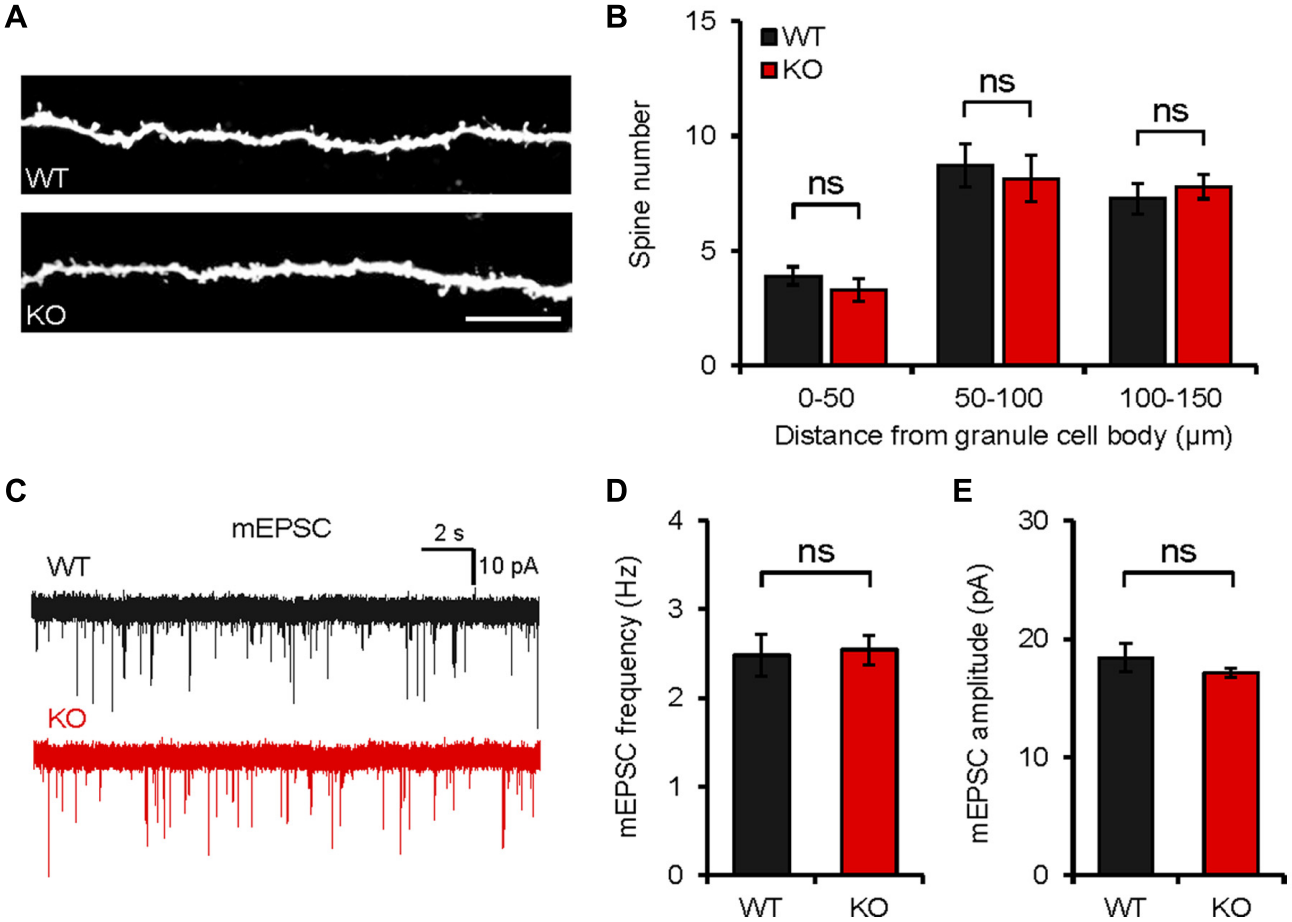
***Neph2^-/-^* mice show normal spine density and spontaneous excitatory transmission in the hippocampal DG. (A,B)** Normal density of dendritic spines in *Neph2^-/-^* DG granule cell dendrites (∼8 weeks), as measured by biocytin infusion into *Neph2^-/-^* slices. (*n* = 11 cells from 5 mice for WT, and 14 cells from 5 mice for KO, ns, not significant, Student’s *t*-test). **(C–E)** Normal frequency and amplitude of mEPSCs in *Neph2^-/-^* DG granule cells (8 weeks). (*n* = 16, 5 for WT, and 17, 5 for KO, ns, not significant, Student’s *t*-test).

In order to determine whether the behavioral changes are associated with any changes at the synapse level, we first measured dendritic spine density. We found that the number of spines in the dendritic region of the *Neph2^-/-^* DG granule cells (∼8 weeks) was not different from that of WT cells, as measured by biocytin labeling of DG granule cells in acute slices (**Figures 6A,B**).

The frequency or amplitude of miniature excitatory postsynaptic currents (mEPSCs) in *Neph2^-/-^* DG granule cells (∼8 weeks) were not different between genotypes (**Figures 6C–E**), consistent with the normal dendritic spine density, and suggesting that Neph2 deletion has little effect on excitatory synaptic density and synaptic content of AMPA receptors. It should be pointed out, however, that we did not measure spine density and synaptic transmission in other parts of the brain, which may be more relevant to the moderate hyperactivity and defective novel object preference observed in *Neph2^-/-^* mice.

## Discussion

In the present study, we have found that Neph2 deletion in mice leads to moderate hyperactivity in a familiar environment and impaired object recognition memory.

These mild behavioral phenotypes of *Neph2^-/-^* mice might be attributable to compensatory upregulations of Neph2-related proteins such as Neph1 or Neph3. However, our unpublished *in situ* hybridization data indicate that Neph2 is strongly expressed in mouse brains, whereas Neph1 and Neph3 mRNAs are almost undetectable, although we cannot exclude such a possibility. Alternatively, it may be that we did not test the behaviors associated other brain regions with high Neph2 expression such as the cerebellum.

*Neph2^-/-^* mice display hyperactivity in the familiar environment but normal locomotor activity in the novel environment. What might be the reasons for this difference? The novel open field arena, which is likely a threatening and stressful stimulus to mice (Walsh and Cummins, 1976; Choleris et al., 2001), may suppress the hyperactivity of *Neph2^-/-^* mice observed in a familiar and completely dark home-cage environment. Alternatively, it could be the higher light intensity in the open field arena (∼20 lux), which is, although dim, certainly brighter than the complete darkness in the home-cage environment. However, additional open field experiment performed in complete darkness did not yield any difference between genotypes. Anxiety in the open field is a less likely possibility because *Neph2^-/-^* mice spent normal amount of time in the center region of the open-field arena, and exhibited normal levels of anxiety-like behaviors in the elevated plus maze test. Although further details remain to be determined, given that many transgenic mouse lines often display strong hyperactivity in both novel and familiar environments, *Neph2^-/-^* mice seem to have a mild hyperactivity.

*Neph2^-/-^* mice display impaired novel object recognition memory but display normal performances in other types of learning and memory tests, including spatial learning/memory in the Morris water maze, fear conditioning, fear extinction, and pattern separation. Although object recognition memory is thought to involve several subregions of the medial temporal lobe such as the hippocampus and perirhinal cortex, accumulating evidence, although controversial, indicate that the perirhinal cortex plays a critical role (Winters et al., 2008). For instance, a clear double dissociation has been reported in studies where a neurotoxic lesion of the perirhinal cortex in rats impairs novel object recognition but leaves spatial memory unimpaired, whereas a hippocampal lesion impairs spatial memory but not novel object recognition (Winters et al., 2004; Forwood et al., 2005). Alternatively, however, *Neph2^-/-^* mice may have changes in the hippocampus, for instance, at early developmental time points. It is also possible that *Neph2^-/-^* mice may have undetected changes in other hippocampus-dependent behaviors.

Neph2 has been associated with ID, cognitive delays associated Jacobsen syndrome, and ASDs (Bhalla et al., 2008; Guerin et al., 2012; Talkowski et al., 2012; Cheng et al., 2013). Specifically, a chromosomal translocation disrupting the *KIRREL3* gene, together with the *CDH15* gene, was found in a 56-years-old female with severe ID (Bhalla et al., 2008). A subsequent search of mutations in the two genes in a large cohort of 647 unrelated patients with ID revealed three non-synonymous missense mutations in the *KIRREL3* gene in patients with mild to severe ID (R40W, R336Q, and V731F; Bhalla et al., 2008). In addition, a small (∼2.9 Mb) interstitial deletion at chromosome 11q24.2–q24.3 including the *KIRREL3* gene was found in a 4-years-old girl with delayed language development and difficulties in social interaction and eye contact (Guerin et al., 2012). Lastly, a translocation breakpoint located ∼40 kb upstream of the *KIRREL3* gene that reduces the expression of the gene by a positional effect was found in a 7-years-old girl with deficits in attention, spatial coordination, and speech (Talkowski et al., 2012). Whether these symptoms are associated, in any meaningful ways, with the phenotypes of *Neph2^-/-^* mice (hyperactivity and recognition memory deficit) remains to be further studied.

## Conclusion

Our study indicates that *Neph2* deletion in mice leads to moderate hyperactivity in a familiar environment and defective novel object preference.

## Author Contributions

S-YC performed multiple *in vivo* experiments including mouse characterization, immunohistochemistry, spine analysis, and behavioral experiments; KH performed immunoblot experiments; TC generated *Neph2^-/-^* mice; HP, DL, and WC performed mEPSC experiments; RK and YC performed learning and memory experiments; M-HK performed pattern separation experiments; KS and EK supervised the project and wrote the manuscript.

## Conflict of Interest Statement

The authors declare that the research was conducted in the absence of any commercial or financial relationships that could be construed as a potential conflict of interest.

## Supplementary material

FIGURE S1 **Expression patterns of Neph2 in the brain (sagittal sections). (A–E)** Expression patterns of Neph2 determined by EGFP staining of the sagittal sections of the *Neph2^-/-^* brain (8 weeks). **(A–E)** lateral to medial sections. Scale bar, 1 mm.

FIGURE S2 **Expression patterns of Neph2 in the brain (coronal sections). (A–D)** Expression patterns of Neph2 determined by EGFP staining of the coronal sections of the *Neph2^-/-^* brain (8 weeks). **(A–D)** rostral to caudal sections. Scale bar, 1 mm.

TABLE S1 **Statistical details of the results**.
