# Supplementary figures and images for: Mice lacking the synaptic adhesion molecule Neph2/Kirrel3 display moderate hyperactivity and defective novel object preference
Source: Front Cell Neurosci. 2015 Jul 28;9:283. doi: 10.3389/fncel.2015.00283 (PMC4517382; doi:10.3389/fncel.2015.00283)

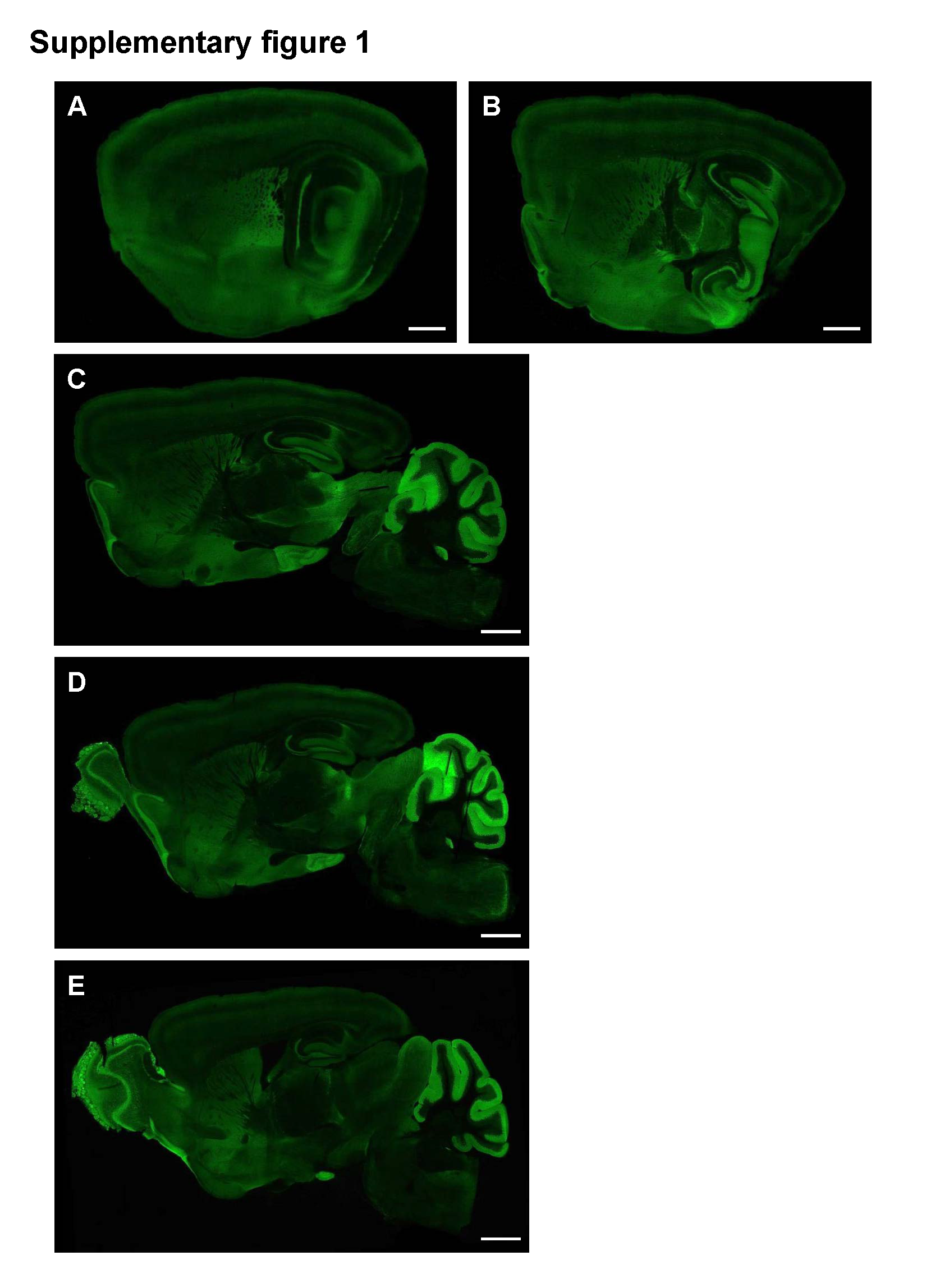

Supplement: FIGURE S1 — Expression patterns of Neph2 in the brain (sagittal sections). (A–E) Expression patterns of Neph2 determined by EGFP staining of the sagittal sections of the Neph2-/- brain (8 weeks). (A–E) lateral to medial sections. Scale bar, 1 mm. [file Image_1.TIFF]

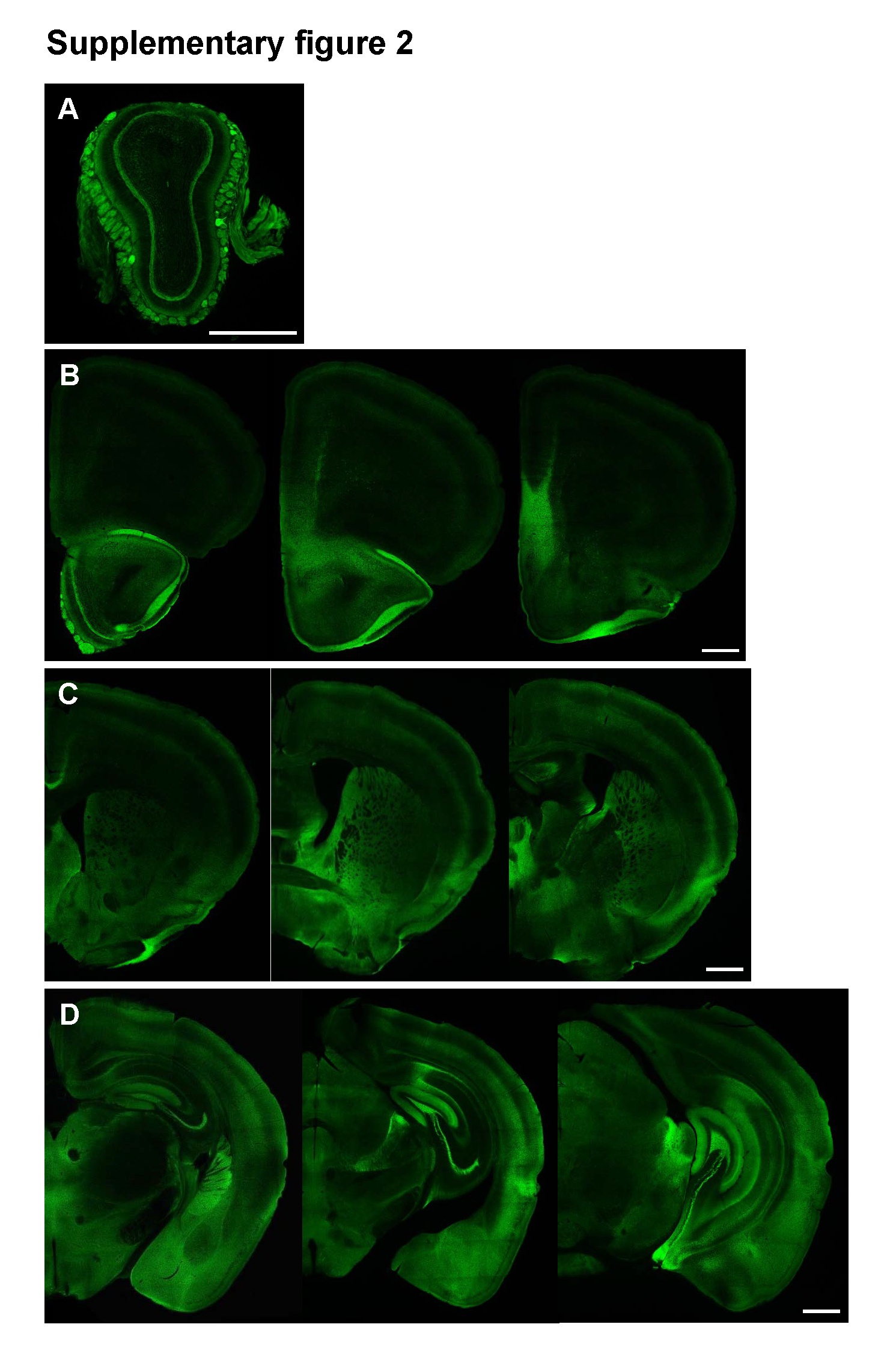

Supplement: FIGURE S2 — Expression patterns of Neph2 in the brain (coronal sections). (A–D) Expression patterns of Neph2 determined by EGFP staining of the coronal sections of the Neph2-/- brain (8 weeks). (A–D) rostral to caudal sections. Scale bar, 1 mm. [file Image_2.TIFF]
