# Supplementary material for: Mice lacking the synaptic adhesion molecule Neph2/Kirrel3 display moderate hyperactivity and defective novel object preference
Source: Front Cell Neurosci. 2015 Jul 28;9:283. doi: 10.3389/fncel.2015.00283 (PMC4517382; doi:10.3389/fncel.2015.00283)
Supplement: TABLE S1 — Statistical details of the results. [file Table_1.PDF]

Supplementary Table S1

Figure		Assay performed	Comparision	Mouse Age	n	Parameter	Descriptive statistics (Average ± SEM)	Statistical test and Significance
2	A	Western blot	WT-KO	3 weeks	WT = 4, KO = 4 (mice)	KO expression level nomalized by α-Tubulin (compared to WT)	PSD-95 = 0.865 ± 0.034; PSD-93 = 0.825 ± 0.049; SAP97 = 0.862 ± 0.063; SAP102 = 0.846 ± 0.064; Synapsin I = 1.008 ± 0.153; Synphy = 1.028 ± 0.227; CaMKIIα/β = 0.840 ± 0.089; CASK = 0.836 ± 0.059	Student's t-test; PSD-95, p = 0.150, t(6) = 1.650, two-tailed; PSD-93, p = 0.062, t(6) = 2.287, two-tailed; SAP97, p = 0.274, t(6) = 1.202, two-tailed; SAP102, p = 0.134, t(6) = 1.729, two-tailed; Synapsin I, p = 0.969, t(6) = 0.043, two-tailed; Synphy, p = 0.928, t(6) = 0.093, two-tailed; CaMKIIα/β, p = 0.307, t(6) = 1.115, two-tailed; CASK, p = 0.054, t(6) = 2.384, two-tailed
	B						GluN2A = 0.851 ± 0.062; GluN2B = 0.960 ± 0.021; GluA1 = 1.120 ± 0.099; GluA2 = 0.875 ± 0.084; mGluR5 = 0.944 ± 0.091	Student's t-test; GluN2A, p = 0.515, t(6) = 0.690, two-tailed; GluN2B, p = 0.893, t(6) = 0.140, two-tailed; GluA1, p = 0.547, t(6) = 0.6368, two-tailed; GluA2, p = 0.285, t(6) = 1.172, two-tailed; mGluR5, p = 0.630, t(6) = 0.5061, two-tailed
	C						PAK1/3 = 1.109 ± 0.191; p-PAK1/3 = 1.163 ± 0.100; LIMK1 = 0.881 ± 0.118; p-LIMK1 = 0.889 ± 0.057; Cofilin = 1.219 ± 0.187; p-Cofilin = 0.923 ± 0.062	Student's t-test; PAK1/3, p = 0.610, t(6) = 0.537, two-tailed; p-PAK1/3, p = 0.159, t(6) = 1.608, two-tailed; LIMK1, p = 0.527, t(6) = 0.670, two-tailed; p-LIMK1, p = 0.422, t(6) = 0.860, two-tailed; Cofilin, p = 0.293, t(6) = 1.151, two-tailed; p-Cofilin, p = 0.719, t(6) = 0.376, two-tailed
3	A	Open-field test at ~20 lux	WT-KO	8-16 weeks	WT = 8, KO = 9 (mice)	Distance moved (m)	WT 10 min = 48.114 ± 5.268, 20 min = 36.203 ± 5.249, 30 min = 32.905 ± 2.863, 40 min = 32.715 ± 3.994, 50 min = 28.123 ± 2.952, 60 min = 29.541 ± 3.708;<br>KO 10 min = 53.946 ± 4.835, 20 min = 36.836 ± 2.812, 30 min = 36.123 ± 2.425, 40 min = 37.046 ± 2.764, 50 min = 35.096 ± 1.515, 60 min = 31.628 ± 1.535	Two-way ANOVA, Repeated measures; Interaction F(5, 75) = 0.72, p = 0.6089, Neph2 F(1, 15) = 0.83, p = 0.3778, Time F(5, 75) = 28.77, p < 0.0001, Bonferroni's post-test, 10 min p > 0.05, 20 min p > 0.05, 30 min p > 0.05, 40 min p > 0.05, 50 min p > 0.05, 60 min p > 0.05
	B					Total distance moved (m)	WT = 207.600 ± 22.062, KO = 230.675 ± 13.789	Student's t-test; WT vs KO, p = 0.3778, t(15) = 0.908, two-tailed
	C					Distance moved (m)	WT 1 min = 6.003 ± 0.575, 2 min = 5.842 ± 0.058, 3 min = 5.300 ± 0.593, 4 min = 5.116 ± 0.580, 5 min = 4.995 ± 0.546, 6 min = 4.594 ± 0.588, 7 min = 4.355 ± 0.549, 8 min = 4.164 ± 0.517, 9 min = 4.067 ± 0.462, 10 min = 3.676 ± 0.613 ;<br>KO 1 min = 6.398 ± 0.578, 2 min = 6.077 ± 0.569, 3 min = 5.897 ± 0.540, 4 min = 5.711 ± 0.480, 5 min = 5.520 ± 0.395, 6 min = 5.087 ± 0.450, 7 min = 5.010 ± 0.495, 8 min = 4.896 ± 0.465, 9 min = 4.650 ± 0.60, 10 min = 4.611 ± 0.676	Two-way ANOVA, Repeated measures; Interaction F(9, 135) = 0.39, p = 0.9368, Neph2 F(1, 15) = 0.67, p = 0.3778, Time F(9, 135) = 19.96, p < 0.0001, Bonferroni's post-test, 1 min p > 0.05, 2 min p > 0.05, 3 min p > 0.05, 4 min p > 0.05, 5 min p > 0.05, 6 min p > 0.05, 7 min p > 0.05, 8 min p > 0.05, 9 min p > 0.05, 10 min p > 0.05
	D					Total distance moved during the first 10 min (m)	WT = 48.11 ± 5.268, KO = 53.95 ± 4.835	Student's t-test; WT vs KO, p = 0.4266, t(15) = 0.8172, two-tailed
	E					Center time (sec)	WT = 440.1 ± 89.85, KO = 518.7 ± 59.17	Student's t-test; WT vs KO, p = 0.4666, t(15) = 0.7470, two-tailed
	F	Open-field test in complete darkness			WT = 8, KO = 8 (mice)	Distance moved (m)	WT 10 min = 56.619 ± 3.381, 20 min = 46.913 ± 2.560, 30 min = 41.947 ± 4.166, 40 min = 40.997 ± 2.877, 50 min = 36.710 ± 2.882, 60 min = 32.961 ± 3.043;<br>KO 10 min = 62.975 ± 2.985, 20 min = 51.546 ± 3.167, 30 min = 44.652 ± 2.449, 40 min = 39.619 ± 1.178, 50 min = 38.570 ± 2.156, 60 min = 34.528 ± 1.397	Two-way ANOVA, Repeated measures; Interaction F(5, 70) = 0.92, p = 0.4715, Neph2 F(1, 14) = 0.72, p = 0.4090, Time F(5, 70) = 45.17, p < 0.0001, Bonferroni's post-test, 10 min p > 0.05, 20 min p > 0.05, 30 min p > 0.05, 40 min p > 0.05, 50 min p > 0.05, 60 min p > 0.05
	G					Total distance moved (m)	WT = 256.10 ± 15.76, KO = 271.9 ± 9.674	Student's t-test; WT vs KO, p = 0.4090, t(14) = 0.8512, two-tailed
	H					Distance moved (m)	WT 1 min = 7.216 ± 0.355, 2 min = 6.692 ± 0.277, 3 min = 6.579 ± 0.397, 4 min = 5.722 ± 0.416, 5 min = 5.520 ± 0.337, 6 min = 5.212 ± 0.533, 7 min = 5.110 ± 0.454, 8 min = 5.107 ± 0.405, 9 min = 4.893 ± 0.380, 10 min = 4.567 ± 0.361;<br>KO 1 min = 7.563 ± 0.473, 2 min = 6.897 ± 0.373, 3 min = 6.362 ± 0.336, 4 min = 6.883 ± 0.426, 5 min = 5.960 ± 0.259, 6 min = 5.769 ± 0.373, 7 min = 6.056 ± 0.332, 8 min = 6.038 ± 0.301, 9 min = 5.971 ± 0.387, 10 min = 5.477 ± 0.483	Two-way ANOVA, Repeated measures; Interaction F(9, 126) = 1.79, p = 0.0771, Neph2 F(1, 14) = 1.99, p = 0.1806, Time F(9, 126) = 19.52, p < 0.0001, Bonferroni's post-test, 1 min p > 0.05, 2 min p > 0.05, 3 min p > 0.05, 4 min p > 0.05, 5 min p > 0.05, 6 min p > 0.05, 7 min p > 0.05, 8 min p > 0.05, 9 min p > 0.05, 10 min p > 0.05
	I					Total distance moved during the first 10 min (m)	WT = 56.62 ± 3.381, KO = 62.97 ± 2.985	Student's t-test; WT vs KO, p = 0.1806, t(14) = 1.409, two-tailed
	J					Center time (sec)	WT = 593.5 ± 46.35, KO = 650.9 ± 39.72	Student's t-test; WT vs KO, p = 0.3629, t(14) = 0.9405, two-tailed
	K	Automated 24hr-movement analysis			WT = 7, KO = 9 (mice)	Distance moved (m)	WT 1 hr = 3.694 ± 0.518, 2 hr = 0.835 ± 0.515, 3 hr = 2.404 ± 1.140, 4 hr = 1.026 ± 0.647, 5 hr = 2.151 ± 0.703, 6 hr = 1.426 ± 0.798, 7 hr = 1.706 ± 0.912, 8 hr = 1.709 ± 0.887, 9 hr = 2.278 ± 0.867, 10 hr = 1.232 ± 0.343, 11 hr = 3.193 ± 1.133, 12 hr = 7.310 ± 1.125, 13 hr = 11.953 ± 0.899, 14 hr = 9.871 ± 1.873, 15 hr = 10.581 ± 1.281, 16 hr = 10.659 ± 1.191, 17 hr = 12.367 ± 1.082, 18 hr = 6.230 ± 1.857, 19 hr = 3.510 ± 1.146, 20 hr = 6.707 ± 1.961, 21 hr = 9.639 ± 2.842, 22 hr = 8.726 ± 1.188, 23 hr = 3.147 ± 0.827, 24 hr = 0.585 ± 0.280;<br>KO 1 hr = 4.14 ± 0.902, 2 hr = 1.374 ± 0.694, 3 hr = 1.955 ± 0.645, 4 hr = 2.055 ± 0.653, 5 hr = 1.065 ± 0.716, 6 hr = 2.810 ± 0.844, 7 hr = 2.027 ± 0.753, 8 hr = 3.056 ± 0.763, 9 hr = 3.806 ± 0.715, 10 hr = 2.199 ± 0.902, 11 hr = 4.879 ± 1.337, 12 hr = 4.225 ± 0.636, 13 hr = 11.059 ± 1.083, 14 hr = 13.690 ± 1.277, 15 hr = 16.877 ± 0.963, 16 hr = 13.494 ± 1.558, 17 hr = 9.805 ± 1.878, 18 hr = 9.838 ± 2.509, 19 hr = 10.426 ± 1.862, 20 hr = 6.773 ± 1.547, 21 hr = 13.020 ± 1.426, 22 hr = 11.615 ± 1.676, 23 hr = 4.826 ± 1.658, 24 hr = 2.455 ± 1.482	Two-way ANOVA, Repeated measures; Interaction F(23, 322) = 1.73, p = 0.0216, Neph2 F(1, 14) = 11.58, p = 0.0043, Time F(23, 322) = 22.42, p < 0.0001, Bonferroni's post-test, 15 hr p < 0.05, 19 hr p < 0.05, 1 hr, 2 hr, 3 hr, 4 hr, 5 hr, 6 hr, 7 hr, 8 hr, 9 hr, 10 hr, 11 hr, 12 hr, 13 hr, 14 hr, 16 hr, 17 hr, 18 hr, 20 hr, 21 hr, 22 hr, 23 hr, and 24 hr p > 0.05
	L					Total distance moved (m)	WT = 123.4 ± 7.841, KO = 153.79 ± 6.308	Student's t-test; WT vs KO, p = 0.0031, t(14) = 3.559, two-tailed
	M					Distance moved (m)	WT Light-on = 22.08 ± 2.489, KO Light-on = 30.64 ± 1.714; WT Light-off = 101.3 ± 7.656, KO Light-off = 123.9 ± 5.495	Student's t-test; WT Light-on vs KO Light-on, p = 0.0110, t(14) = 2.927, two-tailed, WT Light-off vs KO Light-off, p = 0.0273, t(14) = 2.464, two-tailed

3	N	Elevated plus maze	WT-KO	8-16 weeks	WT = 6, KO = 8 (mice)	Time spent (sec)	WT Open arm = 70.97 ± 9.562, KO Open arm = 79.75 ± 7.238; WT Closed arm = 160.1 ± 6.992, KO Closed arm = 159.2 ± 7.663	Student's t-test; WT Open arm vs KO Open arm, p = 0.4689, t(12) = 0.7479, two-tailed, WT Closed arm vs KO Closed arm, p = 0.9316, t(12) = 0.087, two-tailed
	O	3-chamber test			WT = 12, KO = 15 (mice)	Preference index (S1–O)	WT = 55.939 ± 4.671, KO = 51.514 ± 2.900	Student's t-test; WT vs KO, p = 0.409, t(25) = 0.8382, two-tailed
	P					Preference index (S2–S1)	WT = 33.075 ± 7.133, KO = 34.833 ± 6.964	Student's t-test; WT vs KO, p = 0.8628, t(25) = 0.1747, two-tailed
	Q	Buried food test			WT = 12, KO = 8 (mice)	Latency (sec)	Food pellet; WT = 18.57 ± 2.739, KO = 12.58 ± 2.922	Student's t-test; WT vs KO, p = 0.1633, t(18) = 1.454, two-tailed
	R						Vanilla cookies; WT = 11.39 ± 1.760, KO = 11.82 ± 1.457	Student's t-test; WT vs KO, p = 0.8617, t(18) = 0.176, two-tailed
	S	Self-grooming test			WT = 7, KO = 7 (mice)	Self-grooming (sec)	WT = 75.237 ± 9.180, KO = 52.229 ± 5.641	Student's t-test; WT vs KO, p = 0.0540, t(12) = 2.135, two-tailed
4	A	Novel object recognition test	WT-KO	8-16 weeks	WT = 12, KO = 15 (mice)	Novel object preference (%)	WT = 60.811 ± 2.758, KO = 50.586 ± 2.852	Student's t-test; WT vs KO, p = 0.0179, t(25) = 2.535, two-tailed
	B	Morris water maze			WT = 9, KO = 9 (mice)	Escape latency (sec)	WT Day 1 = 55.04 ± 3.367, Day 2 = 46.35 ± 5.506, Day 3 = 46.37 ± 4.032, Day 4 = 26.34 ± 3.986, Day 5 = 31.00 ± 4.341, Day 6 = 29.42 ± 5.561, Day 7 = 17.21 ± 3.935, Day 9 = 40.98 ± 4.174, Day 10 = 30.87 ± 3.788, Day 11 = 26.78 ± 4.429;<br>KO Day 1 = 59.23 ± 0.771, Day 2 = 49.64 ± 4.367, Day 3 = 41.23 ± 4.862, Day 4 = 30.59 ± 6.401, Day 5 = 24.96 ± 3.895, Day 6 = 23.54 ± 3.078, Day 7 = 13.48 ± 2.475, Day 9 = 38.85 ± 4.064, Day 10 = 31.35 ± 4.906, Day 11 = 27.15 ± 4.659	Two-way ANOVA, Repeated measures; Interaction F(9, 144) = 0.48, p = 0.8841, Neph2 F(1, 16) = 0.20, p = 0.6603, Training day F(9, 144) = 17.66, p < 0.0001, Bonferroni's post-test, Day 1, Day 2, Day 3, Day 4, Day 5, Day 6, Day 7, Day 9, Day 10, and Day 11 p > 0.05
	C					Quadrant occupancy (%)	WT Target = 40.23 ± 4.402, Opposite = 15.16 ± 3.215, Left = 17.68 ± 2.910, Right = 26.93 ± 4.009;<br>KO Target = 36.09 ± 4.231, Opposite = 18.72 ± 2.862, Left = 19.68 ± 1.804, Right = 25.51 ± 1.466	Two-way ANOVA, Repeated measures; Interaction F(3, 48) = 0.42, p = 0.7408, Neph2 F(1, 16) = 0.00, p = 1.000, Quadrant F(3, 48) = 13.04, p < 0.0001, Bonferroni's post-test, Target p > 0.05, Opposite p > 0.05, Left p > 0.05, Right p > 0.05
	D					Number of crossing	WT = 3.444 ± 0.294, KO = 3.111 ± 0.5122	Student's t-test; WT vs KO, p = 0.5803, t(16) = 0.5644, two-tailed
	E	Contextual fear conditioning			WT = 9, KO = 9 (mice)	Freezing (%)	WT = 32.46 ± 5.432, KO = 38.18 ± 3.525	Student's t-tes; WT vs KO, p = 0.3902, t(16) = 0.8832, two-tailed
	F	Contextual fear extinction					WT Retrieval day 1 = 32.46 ± 5.43, Reterieval day 2 = 21.22 ± 3.80, Retrieval day 3 = 18.68 ± 4.94, Retrieval day 4 = 13.61 ± 3.44, Retrieval day 5 = 16.70 ± 4.27;<br>KO Retrieval day 1 = 38.18 ± 3.53, Retrieval day 2 = 32.38 ± 6.60, Retrieval day 3 = 24.27 ± 5.86, Retrieval day 4 = 14.82 ± 4.29, Retrieval day 5 = 14.25 ± 3.61	Two-way ANOVA, Repeated measures; Interaction F(4, 64) = 1.27, p = 0.291, Neph2 F(1, 16) = 0.66, p = 0.4295, Retrieval day F(4, 64) = 14.51, p < 0.0001, Bonferroni's post-test, Retrieval day 1 p > 0.05, Retrieval day 2 p > 0.05, Retrieval day 3 p > 0.05, Retrieval day 4 p > 0.05, Retrieval day 5 p > 0.05
5	C	Contextual discrimination	WT-KO	8-16 weeks	WT = 10, KO = 10 (mice)	Freezing (%)	WT Day 1 = 1.32 ± 1.02, Day 2 = 31.71 ± 3.70, Day 3 = 64.46 ± 5.80; KO Day 1 = 0.15 ± 0.15, Day 2 = 27.01 ± 2.89, Day 3 = 60.01 ± 3.93	Two-way ANOVA, Repeated measures; Interaction F(2, 36) = 0.15, p = 0.8649, Neph2 F(1, 18) = 1.87, p = 0.1887, Time F(2, 36) = 142.66, p < 0.0001, Bonferroni's post-test, Day 1 p > 0.05, Day 2 p > 0.05, Day 3 p > 0.05
	D					Freezing (%)	WT Context A = 57.652 ± 3.592, Context B = 49.746 ± 4.742;<br>KO Context A = 48.892 ± 2.894, Context B = 44.996 ± 4.415	Two-way ANOVA, Repeated measures; Interaction F(1, 18) = 0.20, p = 0.6568, Neph2 F(1, 18) = 3.82, p = 0.0663, Context F(1, 18) = 1.77, p = 0.2002, Context A p > 0.05, Context B p > 0.05
	E					Discrimination ratio (Context A / Context A+B)	WT Day 6 = 0.502 ± 0.009, Day 7 = 0.542 ± 0.010, Day 8 = 0.560 ± 0.012, Day 9 = 0.607 ± 0.011, Day 10 = 0.621 ± 0.019, Day 11 = 0.669 ± 0.013;<br>KO Day 6 = 0.508 ± 0.011, Day 7 = 0.512 ± 0.014, Day 8 = 0.541 ± 0.009, Day 9 = 0.572 ± 0.017, Day 10 = 0.618 ± 0.013, Day 11 = 0.650 ± 0.012	Two-way ANOVA, Repeated measures; Interaction F(5, 90) = 0.81, p = 0.5431, Neph2 F(1, 18) = 2.68, p = 0.1187, Time F(5, 90) = 45.08, p < 0.0001, Bonferroni's post-test, Day 6 p > 0.05, Day 7 p > 0.05, Day 8 p > 0.05, Day 9 p > 0.05, Day 10 p > 0.05, Day 11 p > 0.05
	G	Radial arm maze			WT = 21, KO =13 (mice)	Correct choice ratio	WT Separation 1 = 0.738 ± 0.066, Separation 2 = 0.595 ± 0.056, Separation 3 = 0.571 ± 0.052;<br>KO Separation 1 = 0.692 ± 0.070, Separation 2 = 0.577 ± 0.096, Separation 3 = 0.577 ± 0.111	Two-way ANOVA, Repeated measures; Interaction F(2, 64) = 0.07, p = 0.9307, Neph2 F(1, 32) = 0.08, p = 0.7788, Difficulties F(2, 64) = 2.67, p = 0.077, Bonferroni's post-test, Separation 1 p > 0.05, Separation 2 p > 0.05, Separation 3 p > 0.05
6	B	Spine analysis	WT-KO	P49–56	WT = 11 cells from 5 mice, KO = 14 cells from 5 mice	Spine number	WT 0-50 μm = 3.909 ± 0.392, 50-100 μm = 8.727 ± 0.944, 100-150 μm = 7.272 ± 0.647;<br>KO 0-50 μm = 2.150 ± 0.664, 50-100 μm = 8.143 ± 1.005, 100-150 μm = 7.786 ± 0.526	Two-way ANOVA, Repeated measures; Interaction F(2, 46) = 0.71, p = 0.499, Neph2 F(1, 23) = 0.08, p = 0.776, Distance from granule cell body F(2, 46) = 44.86, p < 0.0001, Bonferroni's post-test, 0-50 μm p > 0.05, 50-100 μm p > 0.05, 100-150 μm p > 0.05
	D	Electrophysiology (Whole cell voltage clamp)			WT = 16 cells from 5 mice, KO = 17 cells from 5 mice	mEPSC frequency (Hz)	WT = 2.483 ± 0.231, KO = 2.545 ± 0.165	Student's t-test; WT vs KO, p = 0.8294, t(31) = 0.2172, two-tailed
	E					mEPSC amplitude (pA)	WT = 18.43 ± 1.170, KO = 17.12 ± 0.367	Student's t-test; WT vs KO, p = 0.2843, t(31) = 1.089, two-tailed
